# Identification of a large protein network involved in epigenetic transmission in replicating DNA of embryonic stem cells

**DOI:** 10.1093/nar/gku374

**Published:** 2014-05-22

**Authors:** Sergi Aranda, Dorothea Rutishauser, Patrik Ernfors

**Affiliations:** 1 Unit of Molecular Neurobiology, Department of Medical Biochemistry and Biophysics, Karolinska Institute, 17177 Stockholm, Sweden; 2 Proteomics Karolinska, Department of Medical Biochemistry and Biophysics, Karolinska Institute, Stockholm, Sweden

## Abstract

Pluripotency of embryonic stem cells (ESCs) is maintained by transcriptional activities and chromatin modifying complexes highly organized within the chromatin. Although much effort has been focused on identifying genome-binding sites, little is known on their dynamic association with chromatin across cell divisions. Here, we used a modified version of the iPOND (isolation of proteins at nascent DNA) technology to identify a large protein network enriched at nascent DNA in ESCs. This comprehensive and unbiased proteomic characterization in ESCs reveals that, in addition to the core replication machinery, proteins relevant for pluripotency of ESCs are present at DNA replication sites. In particular, we show that the chromatin remodeller HDAC1–NuRD complex is enriched at nascent DNA. Interestingly, an acute block of HDAC1 in ESCs leads to increased acetylation of histone H3 lysine 9 at nascent DNA together with a concomitant loss of methylation. Consistently, in contrast to what has been described in tumour cell lines, these chromatin marks were found to be stable during cell cycle progression of ESCs. Our results are therefore compatible with a rapid deacetylation-coupled methylation mechanism during the replication of DNA in ESCs that may participate in the preservation of pluripotency of ESCs during replication.

## INTRODUCTION

Pluripotent embryonic stem cells (ESCs) are highly proliferative cells that can expand indefinitely. This unlimited expansion is sustained by their self-renewal capacity, which relies on a high fidelity of the genome and the epigenome transmission during deoxyribonucleic acid (DNA) replication (1,2). The self-renewal capacity and the plasticity to differentiate into all the cell types of an adult organism are orchestrated and balanced by a unique protein interaction network. The network is centred by the pluripotent transcription factors OCT4, NANOG and SOX2, which act in a coordinated manner with chromatin modifying complexes (1,3). These complexes include Polycomb repressor complexes (PRC) 1 and 2, BRG1 associated factors (esBAF) complex and the nucleosomal remodelling and deacetylase (NuRD) complex (1,4).

With the aim to elucidate the functionality of these complexes, intensive efforts have been undertaken to understand precisely where these epigenetic complexes are positioned within the genome in ESCs using chromatin immunoprecipitation combined with massive parallel sequencing (CHIP-seq) (5–7). However, less is known on the dynamics of these interactions, in particular during cell cycle progression. This question is especially relevant for ESCs, which display a rapid cell cycle with a shortened G1 phase and a dominant DNA replication phase (2,8).

Very recently, a novel technique was developed to isolate proteins on nascent DNA (iPOND). Using highly proliferative transformed cells, the iPOND technology has enabled the isolation of proteins already known to be associated with the replication fork (9), as well as, in combination with mass spectrometry, the identification of new replication associated factors in HEK293T cells (10,11). Although core replication proteins were consistently identified, such transformed cell lines are predicted to display abnormalities defining the tumour cell state, including alterations of chromatin regulatory proteins. Therefore, results from these studies lend limited insights of replication associated proteins in ESCs which display unique characteristics compared to other cell types such as a high fidelity during replication (12–14), a unique transcriptional protein network within chromatin and a specific epigenetic composition (1,2).

In this study, we undertook the identification of proteins associated with nascent DNA during the DNA replication phase of ESCs. For this purpose, we used an adapted iPOND technology in combination with high-resolution mass spectrometry. Beside the identification of proteins associated to the replisome and found in recent iPOND screens, our data in ESCs showed a marked enrichment of protein complexes involved in chromatin remodelling and modification. Among these protein complexes, HDAC1-containing complexes, such as NuRD, emerged as central in the protein interaction network that participates in the maintenance of the epigenome in the replication fork of ESCs.

## MATERIALS AND METHODS

### Cell culture

R1 mouse ESCs (from A. Nagy, Toronto, Canada) were cultured as described previously (15,16). For the experiments, ESCs were grown for 48 h on tissue culture plates coated with 0.1% gelatin (Sigma) and cultured in serum-free medium (Knockout Dulbecco's modified Eagle's medium [DMEM], 15% Knockout Serum Replacement, 1× non-essential amino acids, 2 mM glutamine, 5 mM HEPES, 0.4 mM 2-mercaptoethanol [Gibco]) supplemented with 1000 U/ml ESGRO Leukemia Inhibitory Factor (Calbiochem). NIH-3T3 cells (Sigma) were cultured in DMEM supplemented with 10% foetal calf serum (Gibco). The cells were transfected (Lipofectamine 2000; Invitrogen) with 30 nM of siRNA directed against mouse HDAC1 or CHD4 (ON-TARGETplus SMARTpool siRNA; Dharmacon) or with a control siRNA (Non-targeting siRNA; Dharmacon) for 48 h. When indicated, valproic acid (1 mM, Sigma), cycloheximide (10 μg/ml, Sigma) and aphidicolin (0.1–1 μg/ml, Sigma) were used. For differentiation, ESCs cells were grown in N2B27 media (DMEM/F12:Neurobasal 1:1, 0.5× N-2 supplement, 1× B-27 serum-free supplement, 3.2 mM 2-mercaptoethanol; all from Gibco) on monolayer for 5 days (17).

### Western blotting

The samples were analysed by western blot as described previously (18), and the data was quantified using Image Lab software (v4.0.1; Bio-Rad). The antibodies are listed in Supplementary Table S3.

### Isolation of proteins on nascent DNA (iPOND)

The cells were pulsed for 10 min with 100 μM of the thymidine analogue, ethynyl deoxyuridine (EdU). For the chase experiments, the pulse was followed by extensive washing with phosphate-buffered saline (PBS) + 100 μM thymidine (Sigma) and incubation in serum-free media with 100 μM thymidine. Subsequently, the cells were fixed in 1% paraformaldehyde (PFA) for 10 min at room temperature (RT) and quenched with 0.125 mM glycine (pH 7) for 5 min at RT. The cells were harvested, pelleted by centrifugation (720 ×*g*, 10 min at 4°C), and lysed in lysis buffer (ChIP Express kit, Active Motif) for 30 min at 4°C. Lysates were passed 10× through a 21-gauge needle, and the nuclei were pelleted by centrifugation (2400 × *g*, 10 min at 4°C), washed with PBS + protease inhibitor cocktail (PIC; Roche), then subjected to Click reaction for 30 min at RT with 0.2 mM Biotin-azide (Invitrogen). The Click reaction is based on organic chemistry reaction by which an organic azide reacts to a terminal acetylene. The nucleotide exposed ethynyl residue of EdU is derivatized by a copper-catalyzed cycloaddition reaction forming a covalent bound between the EdU and the biotin. The nuclei were re-pelleted by centrifugation (2400 × *g*, 10 min at 4°C), washed with PBS + PIC, suspended in shearing buffer (ChIP Express kit, Active Motif) and sonicated (Bioruptor, Diagenode) for 15 min at high intensity (30-s/30-s on/off pulses). The lysates were cleared by centrifugation (20 800 × *g*, 20 min at 4°C), diluted 1:1 with blocking buffer (1% Triton X-100, 2 mM EDTA [pH 8], 150 mM NaCl, 20 mM Tris-HCl [pH 8], 20 mM beta-glycerol phosphate, 2 mM sodium orthovanadate, PIC and 2 mg/ml salmon-sperm DNA [ssDNA]), then incubated with pre-equilibrated Dynabeads M-280 Streptavidin (Invitrogen) for 30 min at 4°C. The beads were washed two times with blocking buffer (without ssDNA) and twice with high-salt blocking buffer (containing 500 mM NaCl). Finally, the beads were suspended in Laemmli buffer. For mass spectrometry (MS) analysis of iPOND samples, 30–40 × 10^6^ ESCs were lysed in 2 ml of Lysis Buffer, incubated in 2 ml of Click reaction buffer, sheared in 2 ml of shearing buffer, incubated with 1 ml of Dynabeads M-280 Streptavidin and resuspended in 200 ml of Laemmli buffer 2×.

### Two-step (IP-iPOND) purification

Cells were trypsinized, pelleted by centrifugation (150 × *g*, 5 min at 4°C) and lysed in hypotonic buffer A (10 mM HEPES [pH 7.9], 10 mM KCl, 1.5 mM MgCl_2_, 0.34 M sucrose, 10% glycerol, 1 mM dithiothreitol [DTT], 10 mM beta-glycerol phosphate, 1 mM sodium orthovanadate and PIC) + 0.1% Triton X-100 for 5 min at 4°C. Nuclei were collected by centrifugation (1300 × *g*, 4 min at 4°C) and washed with PBS. Click reaction was then performed using 0.2 mM Biotin-azide for 30 min at RT. Nuclei were washed with PBS and lysed in buffer B (3 mM EDTA, 0.2 mM EGTA, 1 mM DTT, 10 mM beta-glycerol phosphate, 1 mM sodium orthovanadate and PIC) for 30 min at 4°C and the lysates were sonicated for 10 min at low intensity (30-s/30-s on/off pulses) and cleared by centrifugation (15 000 × *g*, 20 min at 4°C). Two aliquots were taken for DNA extraction and dot-blot analysis, and the remainder was incubated overnight with 10 μg normal goat IgG or goat anti-HDAC1 antibody. Dynabeads Protein G (50 μl; Invitrogen) that were pre-blocked with PBS + 2 mg/ml ssDNA were added to the samples for 1 h at RT. Beads were washed 4× with PBS + 2 mg/ml ssDNA, and the immunocomplexes were eluted with 50 μg of anti-HDAC1 competitor peptide (Santa Cruz Biotechnology) for 2 h at RT. Eluates were then incubated with Dynabeads M-280 Streptavidin for 30 min at RT, washed 3× with PBS + 2 mg/ml ssDNA, and suspended in Laemmli buffer.

Detailed methods, including FACS analyses, immunofluorescence, dot-blot analysis, DNA purification, immunoprecipitation, antibodies (Supplementary Table S3), mass spectrometry procedures and procedures for data analysis can be found in Supplementary Experimental Materials and Methods.

## RESULTS

### Efficient purification of proteins associated to nascent DNA in ESCs

Protein–protein interaction and immunolocalization studies have provided remarkable impetus for the identification of several DNA replication components (19–21). However, these strategies have been hampered since they are restricted to specific proteins or complexes and depend on the availability and specificity of antibodies. A recently developed technology to isolate proteins on nascent DNA (iPOND) in an unbiased way overcomes these limitations (9). The iPOND technique utilizes the rapid incorporation of the thymidine analogue 5-ethynyl-2′-deoxyuridine during DNA replication, covalent cross-linking between DNA and proteins, the addition of a biotin moiety to the incorporated EdU using mild conditions and streptavidin-biotin affinity to capture sheared EdU-labelled chromatin. The procedure was originally reported to yield the isolation of ∼0.5% of the replication-associated protein PCNA (9). The low efficiency might be a limiting factor which at least partly can be compensated by increasing cell numbers to identify low abundant proteins (10,11). However, the long incubation time with streptavidin-coupled agarose beads of the initial method can result in a high background and the requirement of large number of cells limits the usefulness as a standard biochemical technique.

**Figure 1.**
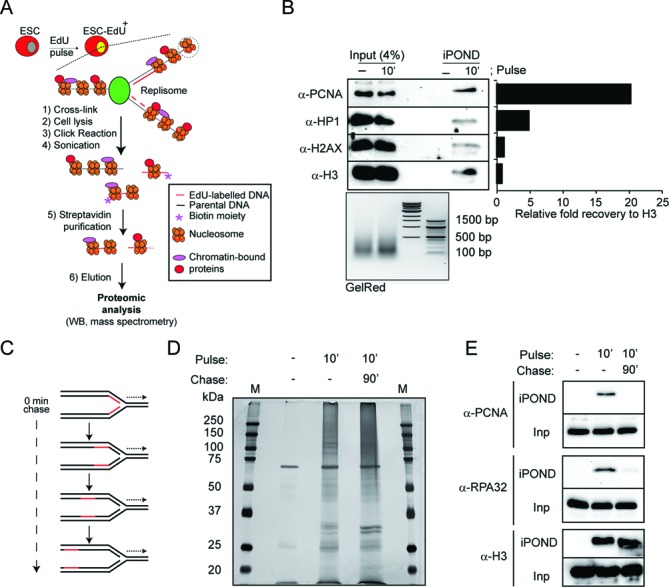
Isolation of proteins on nascent DNA in ESCs. (**A**) Schematic representation of the modified iPOND technique. Cells were incubated with a short pulse of EdU, which was then incorporated into nascent DNA. The DNA and associated proteins were cross-linked (i), cells were lysed (ii), and labelled DNA was conjugated to a biotin group by a Click reaction (iii) and then fragmented by sonication (iv). Labelled DNA fragments were isolated using streptavidin magnetic beads (v) and eluted using Laemmli Buffer (vi). Eluted samples were analysed by western blot or high-resolution mass spectrometry. ESC, embryonic stem cells. ESC−EdU+, embryonic stem cells pulsed with EdU. (**B**) Input and the isolated proteins on nascent DNA (iPOND) from ESCs non-pulsed (−) and pulsed 10 min (10′) with EdU were analysed by western blot using the indicated antibodies. The sonicated DNA was analysed by agarose gel electrophoresis (GelRed). The histogram shows the relative fold recovery of the indicated proteins by iPOND. (**C**) Schematic representation of EdU labelled (red) replication fork progression after a short pulse of EdU. (**D**) Nascent DNA was isolated using the modified iPOND technique from ESCs incubated with EdU as indicated. The samples were separated by SDS-PAGE, and the gel was silver stained. Molecular weights (kDa) for the protein marker (M) are indicated. (**E**) ESCs were pulsed with EdU for 10 min. When indicated, pulsed cells were chased for 90 min after washing out the EdU from the media. The input (Inp) and the iPOND samples were analysed by western blot using the indicated antibodies.

To increase the purification efficiency, we set up conditions to use streptavidin-coupled superparamagnetic beads instead of streptavidin-coupled agarose beads (Figure 1A). This modification not only reduced the required incubation time (from overnight to 30 min) and decreased non-specific interactions, but also resulted in a total recovery of around 4% of total PCNA after a short EdU pulse of 10 min (Figure 1B, Supplementary Figure S1A). The increased efficiency in the isolation of nascent DNA was also confirmed by the purification of chromatin-bound proteins, like heterochomatin protein 1 (HP1), the histone variant H2AX or histone H3 (Figure 1B). Of note, the relative enrichment of the replication associated protein PCNA was greater than that of the chromatin-related proteins, supporting an efficient capture of specifically nascent DNA (Figure 1B).

In order to monitor the amount of EdU-labelled DNA, we introduced a sensitive dot blot assay to allow for its quantification from sheared DNA samples (Supplementary Figure S1B, S1C and S1D). Accordingly, this dot blot assay was systematically used in all the purifications allowing the comparison of, not only untreated pulse and pulse-chased cells, but also when cells were pulsed in different experimental conditions, therefore, increasing the versatility of the technology. Combined, these modifications of the iPOND technology should open for the method as a standard biochemical technique to study chromatin composition at replication sites.

### Identification of the protein network associated to nascent DNA in ESCs

With the aim for an unbiased identification of all protein complexes enriched specifically at nascent DNA in ESCs, we used the modified iPOND technology in combination with high-resolution LTQ-Orbitrap mass spectrometry. To differentiate between proteins specifically associated with nascent DNA, such as the sliding clamp protein PCNA, and constitutive chromatin-bound proteins, such as histone proteins, we compared proteins isolated after a short EdU pulse of 10 min, when the recently replicated DNA is labelled, with proteins isolated after a short EdU pulse followed by a 90-min chase when the replication fork is expected to progress, leaving behind the labelled DNA (Figure 1C). Comparison of protein samples in silver stained polyacrylamide gels loaded with unlabelled and EdU-labelled samples collected either directly after a 10-minpulse or following a 90-min chase showed a high specificity and sensitivity of the technique for isolation of proteins associated with EdU-labelled DNA from ESCs (Figure 1D).

As a validation of our experimental conditions, we used well-known protein markers of both the replication fork and mature chromatin. As shown in Figure 1E, while the replication associated proteins PCNA and RPA32 were efficiently isolated only from pulsed cells, histone H3 was equally purified from both pulse and pulse-chased cells. We also used the level of acetylation of histone H4 at lysines 5 and 12 (H4K5Ac and H4K12Ac, respectively) as a marker, since high levels are characteristic for newly synthetized histones, which are rapidly deacetylated upon deposition (22). Indeed, H4K5Ac and H4K12Ac were significantly enriched in the pulse sample compared to pulse-chase (Supplementary Figure S2A), according with the process of chromatin maturation (22). Consistent with a normal progression of the replication fork, we did not observed any increase in the phosphorylation of H2AX at serine 139 (γH2AX), an early marker of DNA damage, on cells pulsed and pulse-chased with EdU (Supplementary Figure S2B).

**Figure 2.**
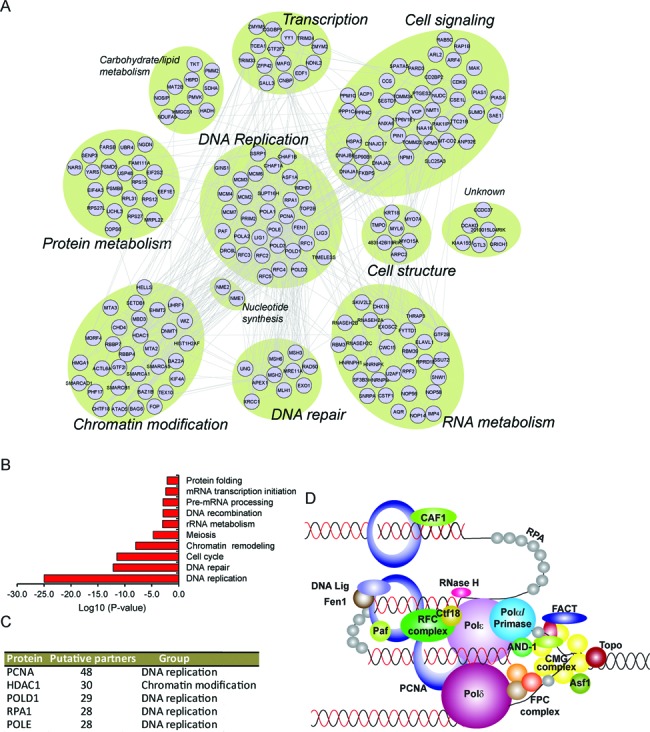
Identification of a protein interaction network associated with nascent DNA in ESCs. (**A**) The protein–protein interaction network of the proteome as defined by STRING analysis. The topology is organized according to functional classification, as indicated. (**B**) Gene ontology (GO) analysis of the biological processes of the proteins identified at nascent DNA. The bars indicate the log_10_ (*P*-value) for the enriched groups. *P*-values represent a modified Fisher Exact Test, EASE Score. (**C**) List of the top five proteins with most interacting partners retrieved from the protein–protein interaction network. (**D**) Schematic depiction of replication-associated factors that were identified in this study. At least one component of each of the indicated multi-protein complexes was present in our data set. Abbreviations: CMG, for CDC45, MCM2–7 helicases and Gins complex; FPC, for fork-protecting complex (containing TIMELESS, TIPIN and CLASPIN proteins); FACT, for Facilitates chromatin transcription complex (containing SSRP1 and SUPT16H); RFC, for replication factor C complex (containing RFC1–5).

Proteins associated with nascent DNA were isolated from gels in four biological replicates and used for identification by high-resolution mass spectrometry. Proteins identified in at least two out of four independent experiments were included for further analysis. A total of 207 proteins were considered as nascent DNA-bound proteins in ESCs since they showed a relative higher enrichment on Mascot Score in the samples pulsed with EdU (Figure 2A and Supplementary Table S1) in comparison with the corresponding pulse-chased samples (Supplementary Table S1). Some of the proteins identified (57 out of 207) have been previously reported to be linked to DNA replication (Supplementary Table S1); in addition, only 44 of the 207 proteins detected in ESCs have been linked to nascent DNA in previous iPOND studies on HEK-293T cells (10,11) (Supplementary Figure S3 and Supplementary Table S1), supporting the existence of a large number of novel proteins with putative roles during DNA replication in ESCs.

To get further insight into the nascent DNA-associated proteins in ESCs, we examined their potential relationships using the STRING database (Figure 2A). We first manually classified the proteins according to the best-known function, resulting in a network with a total of 11 functional clusters that were centered around the DNA replication cluster (Figure 2A). As expected, gene ontology (GO) analysis revealed an enrichment of proteins associated with DNA replication (Figure 2B), and four DNA replication proteins appeared among the top five proteins with most putative partners identified in our data set (Figure 2C).

iPOND mass spectrometry of fibroblasts (NIH 3T3 cells) (Supplementary Table S1) revealed that nascent DNA bound proteins constituting the DNA replication cluster were found to be broadly represented in both ESCs as well as in mouse fibroblasts (Supplementary Figure S4). The cluster included components or subunits identified for virtually all the replisome activities, including the helicase complex, topoisomerase, DNA polymerases, the fork-protecting complex, the sliding clamp protein (PCNA), the clamp loader complex (RFC), the RPA complex, nucleases, ligases, RNases and histone chaperones (Figure 2D and Supplementary Figure S4). These results confirm a high conservation of the core replication module between cell types and further validate the sensitivity of our methodology to isolate replication-associated proteins.

GO term analysis of the proteins identified in ESCs profiling also showed a significant enrichment of proteins annotated to functionally affect cell cycle progression and/or genomic stability (Figure 2B). To further explore these associations, pair-wise analysis was carried out on data from several genome-wide siRNA studies performed in HeLa and U2OS cells for the identification of genes involved in cell division, genomic stability and in ESCs for identification of pluripotency genes (Table 1). From the 207 proteins, 45 and 28 were previously found to affect cell cycle progression and/or genomic stability in siRNA screens, respectively (Table 1). However, only proteins affecting the progression during S-phase were significantly enriched in the iPOND-MS data set (Table 1), supporting a functional link between the proteins identified and the replication of cells. Most strikingly, a number of proteins were also found in our data set that participates in the control of ESC pluripotency (Table 1). These significantly enriched proteins include among others the YY1 DNA-binding protein that mediates DNA targeting of the chromatin modifying polycomb repressive complex 2 (PRC2) (23,24), the catalytic subunit of the protein phosphatase 4 (PPP4C) that affect histone acetylation (25), and the NuRD complex subunit methyl-CpG-binding domain 3 (MBD3) that regulates transcriptional heterogeneity in ESCs (7). Hence, these results reveal that synthesis of new chromatin and the maintenance of pluripotency during cell division might be intimately linked.

**Table 1.** Pair-wise comparison between iPOND-MS data set and genetic screens designed for identification of proteins involved in pluripotency, cell cycle progression and genome instability.

Screening	Analysis	Total genes screened		Hits	Specie	Overlaping hits	Genes	*P*-value	Significance
Pluripotency	Genome-wide^1^	16 683		148	Mouse	7	Yy1, Pcna, Ppp4c, Cdk9, Rprd1b, Ssu72, Mbd3	0.0007	***
	Genome-wide^2^	21 121		173	Human homologs	5	Yy1, Xrcc1, Cggbp1, Eif2s2, Edf1	0.0333	*
	Chromatin factors^3^	1008		68	Mouse	11	Dhx15, Mcm4, Mcm6, Ssrp1, Mcm7, Aqr, Eif4a3, Supt16h, Skiv2l2, Chaf1b, Mbd3	0.5443	ns
	Transcription and chromatin factors^4^	2219		43	Mouse	4	Setdb1, Chaf1b, Chaf1a, Mbd3	0.9957	ns
Cell cycle	Genome-wide^5^	17 828	G0/1 arrest	721	Human homologs	10	Fyttd1, Gtf2b, Sestd1, Rpf2, Rnaseh2c, Mlh1, Hnrnpm, Hsp90b1, Dnajb6, Arf4	0.8831	ns
			S arrest	215	Human homologs	10	Pcna, Rpa1, Pola1, Pole, Timeless, Rpl31, Rbbp4, Nop14, Chaf1a, Atad5	0.0001	***
			G2 arrest	126	Human homologs	4	Sf3b3, Ppp4c, Kif4, Gtl3	0.1333	ns
			Cell division defect	289	Human homologs	6	Zfp42, U2af1, Rprd1b, Eif4a3, Hnrnpk, Hnrnph1	0.3289	ns
	Genome-wide^6^	24 373	G1 arrest	304	Human homologs	2	Rprd1b, Rbbp7	0.9002	ns
			S arrest	83	Human homologs	2	Pola1, Cd2bp2	0.3845	ns
			S+G2M arrest	71	Human homologs	6	Rpa1, Chaf1a, Rps27l, Nosip, Fkbp5, Cwc15	0.0001	***
			G2M arrest	696	Human homologs	9	Rfc2, Msh6, Sumo1, Snw1, Rps15, Rps12, Psmd5, 2010015L04Rik, Cops6	0.3535	ns
Genome Instability	Genome-wide^7^	21 000		2402	Human homologs	28	Rpa1, Sumo1, Snw1,Rps15, Rps12, 2010015L04Rik, Aqr, Pola2, Sf3b3, Timeless, Eif4a3, Fam111a, Rfc2, Pmvk, Ubr4, Elavl1, Vcp, Eif2s2, Hdac1, Gins1, Hist1h2af, Cwc15, Psmd5, Smarca1, Tex10, Hnrnpk, Fop, Arpc2	0.407	ns

The type of screening, the total number of genes screened and the number of hits identified in each study are indicated. The number of overlapping genes present in the iPOND-MS data set and the probability resulting from random sampling (Chi-square test with Yates’ correction test) are indicated.

References: ^1^Genes Dev.2009 Apr 1;23(7):837-48., ^2^Nature.2010 Nov 11;468(7321):316-20., ^3^Cell.2008 Jul 11;134(1):162-74., ^4^Nature.2010 Sep 23;467(7314):430-5., ^5^Nat Cell Biol.2007 Dec;9(12):1401-12., ^6^Proc Natl Acad Sci U S A.2006 Oct 3;103(40):14819-24, ^7^Mol Cell.2009 Jul 31;35(2):228-39.

### Differential recruitment of MMR proteins at nascent DNA

**Figure 3.**
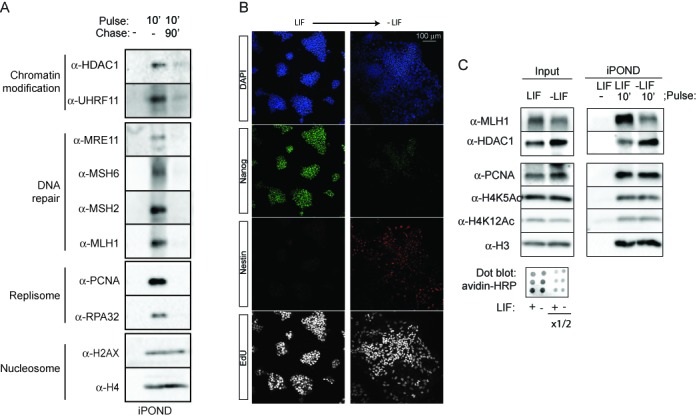
Differential recruitment of MMR proteins at nascent DNA.(**A**) ESCs were incubated with EdU as indicated. The iPOND samples were analysed by western blot using the indicated antibodies. Functional clusters according to Figure 2 are indicated. (**B**) ESCs were differentiated in N2B27 media and stained with anti-Nanog and anti-Nestin antibodies. The cells in S-phase were labelled by EdU and visualized using the Click reaction. (**C**) ESCs and differentiated cells were incubated with EdU as indicated. The input and the iPOND samples were analysed by western blot using the indicated antibodies. The amount of EdU-labelled DNA was analysed by dot-blot.

In addition to the DNA replication cluster, the protein network at nascent DNA in ESCs encompasses 10 functional clusters (Figure 2A). The DNA repair cluster included proteins such as members of the mismatch repair system (MMR: MHS2, MSH6, MLH1) and the double-strand break repair protein MRE11A. These proteins were confirmed to be enriched at nascent DNA in ESCs by western blot with specific antibodies (Figure 3A). The enrichment on MMR proteins at nascent DNA concurs with previous studies in yeast (26) and the more recent iPOND mass-spectrometry data on HEK-293T cells (10,11). These results suggest that DNA repair systems could be an integral part of the replication machinery across different cell types, although analysis of the mutation frequency (12) and total proteins levels between fibroblasts and ESCs (Supplementary Figure S5) indicate quantitative differences in MMR system activity in ESCs and somatic cells.

In order to enable the comparison between different cell types, ESCs were differentiated by few days of LIF (Leukemia inhibitory factor) withdrawal. We observed that the repair protein MLH1 was markedly decreased at nascent DNA upon differentiation, while similar amounts of PCNA, histone H3 and newly assembled histone H4 (H4K5Ac and H4K12Ac) were isolated from ESCs and differentiated ESCs samples (Figure 3B and C). This result confirms that the association to replication sites of the proteins not belonging to the DNA replication core can be regulated in a cell type-dependent manner. Moreover, the results indicate that the high DNA replication fidelity observed in ESCs in contrast to differentiated cells (12–14) could be explained molecularly by a distinctive recruitment of MMR proteins at nascent DNA.

### The HDAC1–NuRD complex is enriched at nascent DNA in ESCs

The class I histone deacetylase protein HDAC1 was among the top five proteins with most known interacting partners identified by STRING analysis in the ESC screen (Figure 2C). The protein was also identified in fibroblasts (Supplementary Table S1) and confirmed to be present in nascent DNA not only in ESCs but also in differentiated ESCs (Figure 3A and 3C), indicating that it could play a central role in chromatin organization during replication.

**Figure 4.**
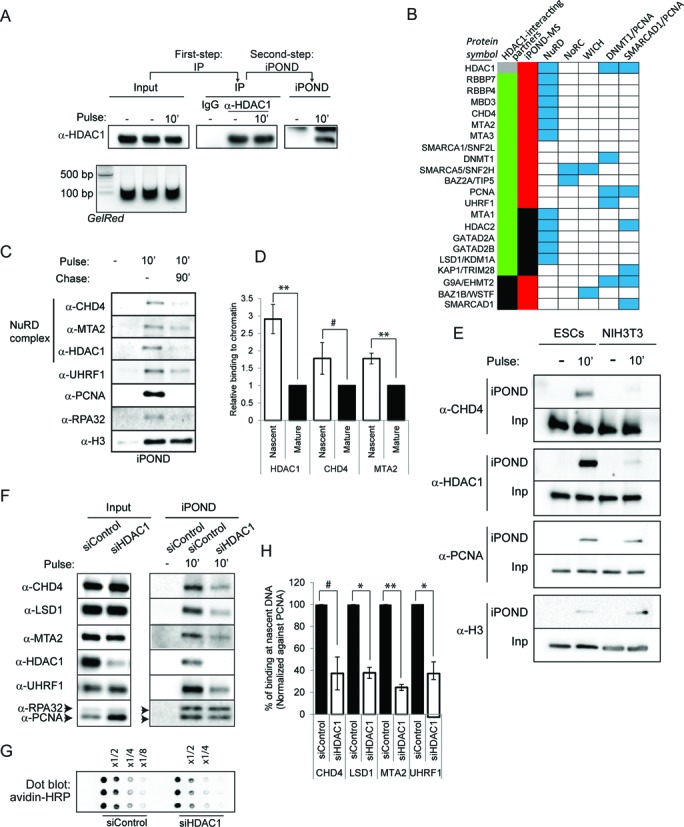
The HDAC1–NuRD complex is enriched at nascent DNA in ESCs. (**A**) Tandem purification procedure. Extracts from ESCs pulsed with EdU were immunoprecipitated first using an anti-HDAC1 antibody. The precipitates were eluted with a competitive peptide and nascent DNA purified by iPOND. The samples were analysed by western blot using the indicated antibody. Note that the sonication yielded small DNA fragments (GelRed). (**B**) Table indicating the proteins identified as HDAC1-interacting partners by the tandem purification procedure and mass spectrometry and/or western blot. The presence of each component in the iPOND-MS analysis is indicated. Green indicates identified proteins using IP-iPOND, red indicates identified proteins by iPOND and blue the respective chromatin-modifying complex they associate with. (**C**) ESCs were incubated with EdU as indicated. The iPOND samples were analysed by western blot using the indicated antibodies. (**D**) Histogram showing quantitative measurements of the relative binding to chromatin for the indicated proteins normalized by the levels of histone H3 (average ± SEM, biological replicates *n* = 4, **P < 0.01, ^#^*P* = 0.1, *t*-test). (**E**) ESCs and NIH-3T3 were pulsed with EdU for 10 min and samples were normalized according to the normalization procedure outlined in (Supplementary Figure S1). Input (Inp) and iPOND samples were analysed by western blot using the indicated antibodies. (**F**) ESCs were transfected with a control siRNA (siControl) or siRNA directed against HDAC1 (siHDAC1). Nascent DNA was then isolated by iPOND technique. The input and iPOND samples were analysed by western blot using the indicated antibodies. (**G**) Dot-blot from sheared samples transfected with control siRNA (siControl) or siRNA directed against HDAC1 (siHDAC1). (**H**) Histogram showing the percentage of binding at nascent DNA for the proteins indicated normalized by PCNA (average ± SEM, biological replicates *n* = 2, ^#^*P* = 0.1, *P < 0.05, **P < 0.01, *t*-test).

HDAC1 is known to be a part of several multiprotein complexes, including the chromatin remodelling complexes NuRD, SIN3 and CoREST (27), which have been classically associated to the regulation of transcription in many mammalian cell types. To address if HDAC1 specific protein complexes are associated to nascent DNA in ESCs, we used a two-step purification protocol to isolate specifically HDAC1-containing nucleosomes from nascent DNA in native conditions combined with high-resolution mass spectrometry for the identification of the bound proteins (Figure 4A). ESCs pulsed with EdU for 10 min were collected and lysed in non-fixative conditions. Cell extracts were sonicated to obtain chromatin with a size comparable to mononucleosomes (Figure 4A, GelRed). The extracts were used for affinity purification with a specific anti-HDAC1 antibody. The purified immunocomplexes were eluted with a competitor peptide and re-purified with streptavidin magnetic beads using the modified iPOND technology. Analysis of the immunoprecipitation-iPOND (IP-iPOND) by mass-spectrometry showed the association of HDAC1 with all the components of the NuRD complex (28), including the ATPase CHD4, lysine-demethylase 1 (LSD1), MBD3, the metastasis-associated gene 1, 2 and 3 (MTA1–3) proteins, and retinoblastoma-binding proteins 4 and 7 (RBBP4 and RBBP7) and the GATA zinc finger domain-containing protein 2A (GATAD2A and GATAD2B) (Figure 4B and Supplementary Table S2). The interaction of HDAC1 with the NuRD components at nascent DNA was confirmed by IP-iPOND followed by western blot analysis with specific antibodies (Supplementary Figure S6).

These previous data identifies all NuRD complex proteins by iPOND as well as HDAC1 IP-iPOND in ESCs, suggesting that NuRD is enriched in ESCs. The enrichment at nascent DNA of the key subunits of the NuRD complex in ESCs was confirmed by iPOND and western blot (Figure 4C and D). Interestingly, the key subunits of NuRD complex, CHD4 and HDAC1, displayed a remarkably greater association with nascent DNA in ESCs, as compared to fibroblasts (NIH3T3 cells) (Figure 4E). Isolation of equivalent amounts of nascent DNA by iPOND was confirmed by the detection of comparable amounts of PCNA and histone H3 (Figure 4E). These results suggest a cell-type dependent regulation of NuRD complex association with nascent DNA. The functional interaction of the complex was further confirmed by the knockdown of HDAC1 using small interfering RNA (siRNA). Depletion of HDAC1 did not affect the rate of EdU incorporation in ESCs (Figure 4G), consistent with previous studies showing that the rate of replication in not substantially different between control and HDAC1 knockout ESCs (29). Furthermore, we did not observe changes in the total amount of NuRD components (Figure 4F, input). However, the recruitment of NuRD components to nascent DNA was severely compromised upon HDAC1 depletion (Figure 4F, iPOND). Quantification of proteins isolated by iPOND and normalized to isolated PCNA confirmed this conclusion (Figure 4H). Combined, these data evidences a recruitment of functional NuRD complex at nascent DNA in ESCs.

Our proteomic data also revealed the association of HDAC1 with other chromatin remodelling complexes that have been shown to play a role in the restoration of transcriptional repressed heterochromatin, such as the NoRC complex (30), the WICH complex (31), the DNMT1/PCNA complex (32) and the SMARCAD1/PCNA complex (33) (Figure 4B). Combined, these data point to HDAC1 as a hub for a protein network at nascent DNA required for epigenome maintenance during replication of ESCs.

### NuRD complex interacts with the hemimethylated DNA-bound protein UHRF1

The iPOND analysis also showed the presence of the epigenetic regulator UHRF1 protein specifically at nascent DNA (Figure 3A) and in the HDCA1 complexes associated with nascent DNA (Figure 4C). The association of these proteins with nascent DNA is functionally linked to fork progression, as revealed by iPOND on ESCs treated by aphidicolin, a reversible inhibitor of the DNA polymerase complex. Acute inhibition of the replication fork progression by aphidicolin reduced association of the NuRD subunits CHD4, HDAC1 and LSD1, as well as UHRF1, while increasing the damage-recognition protein RPA32 (Supplementary Figure S7). The interaction of these proteins appears to be highly dynamic, because when DNA replication fork progression was restored upon aphidicolin removal, the association of the NuRD complex at nascent DNA was normalized (Supplementary Figure S7).

**Figure 5.**
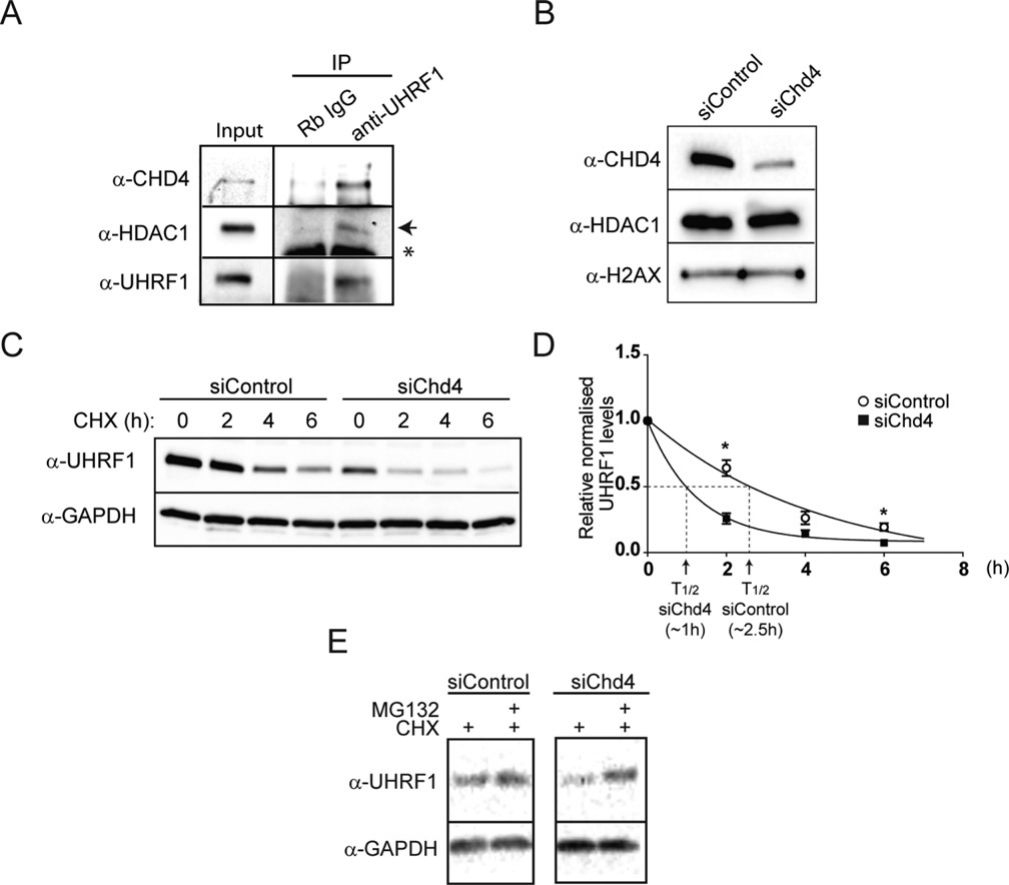
The nucleosomal remodelling and deacetylase (NuRD) complex is associated with the epigenetic regulator UHRF1 in ESCs. (**A**) ESC extracts were immunoprecipitated using an anti-UHRF1 antibody in the presence of ethidium bromide to prevent indirect binding via DNA. Input and immunocomplexes were analysed by western blot. The arrow indicates the specific HDAC1 band and the asterisks point to an unspecific cross-reacting band. (**B**) ESCs were transfected with siControl or CHD4-specific siRNA. The efficiency of the knock-down was analysed by western blot. (**C**) ESCs transfected with siControl or CHD4-specific siRNA were treated with the translational inhibitor cycloheximide (CHX) for indicated times (in hours). Total cell extracts were then analysed by western blot. (**D**) The graph indicates the average of protein expression decay at different time points (average ± SEM, biological replicates *n* = 2, *P < 0.05, *t*-test)). The levels of UHRF1 were normalized against the levels of GAPDH and the time point 0 was set as 1 in each condition. The arrows indicate the estimated half-life for siControl and siCHD4 transfected ESCs. (**E**) ESCs were transfected with siControl or CHD4-specific siRNA and treated with the proteasome inhibitor MG132 in the presence of cycloheximide for 2 h (CHX). Total cell extracts were then analysed by western blot.

UHRF1 was previously shown to bind hemimethylated DNA in association with PCNA at replication sites (32) and to interact with HDAC1 at the p21 gene promoter (34). By RNAi, the recruitment of UHRF1 to the replicating DNA was shown to depend on the presence of HDAC1 (Figure 4F and G). Interestingly, immunoprecipitation of UHRF1 from soluble extracts of ESCs in the presence of ethidium bromide to exclude an indirect DNA-mediated interaction, showed HDAC1 as well as the main component of the NuRD complex, CHD4, in the immunocomplexes (Figure 5A), suggesting that UHRF1 and NuRD are part of the same protein complex. Furthermore, the protein stability of UHRF1 was markedly reduced when the NuRD complex was compromised by CHD4 RNAi, as seen by the estimation of the half-life of UHRF1 in the presence of the translation inhibitor cycloheximide (Figure 5C and D) and the restoration of normal levels in CHD4 siRNA samples in the presence of the proteasomal inhibitor MG132 (Figure 5E). These data provide a functional relationship between NuRD and the replication-associated protein UHRF1 that could participate in re-establishing histone epigenetic marks and regulate chromatin organization following replication fork passage in ESCs.

### Deeply repressed heterochromatin is rapidly restored upon replication fork passage in ESC

New and old histones are rapidly deposited on nascent DNA after replication fork passage (35). New histones need to acquire the same posttranslational modification pattern as the parental in order to maintain the epigenetic code across cell division. However, several reports using cell cycle synchronized HeLa cells conclude that, in contrast to acetylation of new histones, which is rapidly adjusted upon deposition, the methylation of the new histones, including H3K9 trimethylation (H3K9me3), is delayed and not fully restored until the G1 phase of the next cell cycle (36–38). Thus, histone methylation appears uncoupled from replication in HeLa cells. A consequence of this transient unbalance between histone deacetylation and methylation, which normally affects transcription in an opposite ways, may underlie observed oscillations of gene activity across cell cycle phases.

**Figure 6.**
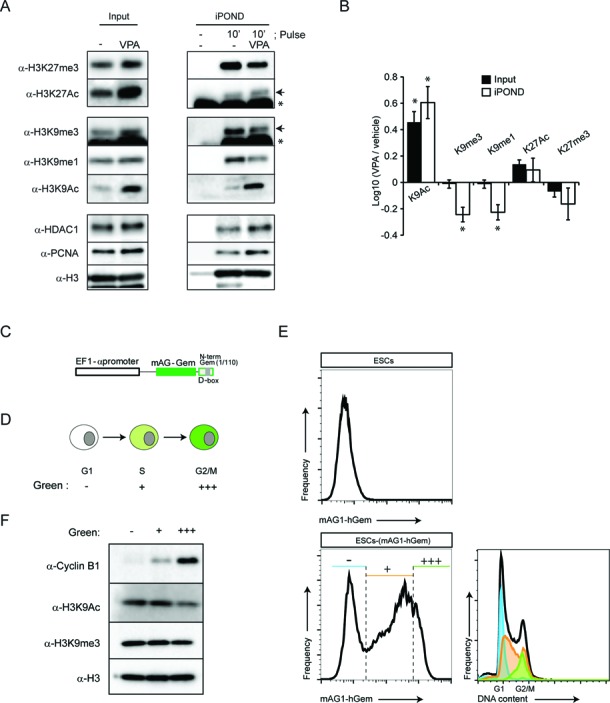
The acetylation and methylation of H3K9 are functionally linked upon replication fork passage in ESC. (**A**) ESCs were incubated with 1 mM VPA for 30 min and labelled the last 10 min with EdU. Input and iPOND samples were analysed by western blot using the indicated antibodies. The arrows indicate specific bands for H3K9me3 and H3K27Ac, the asterisks indicate an unspecific cross-reaction of the antibody. (**B**) Quantification of the average relative enrichment for the repressive histone marks lysine 9 mono- and trimehtylation and lysine 27 trimethylation (K9me1, K9me3 and K27me3, respectively) of histone H3 and its corresponding acetylated forms (K9Ac and K27Ac), normalized by the levels of PCNA (average ± SEM, biological replicates *n* = 3; *P < 0.05, *t*-test). (**C**) Scheme of FUCCI reporter mAG1-hGem transfected in ESCs. (**D**) Coloured code scheme for the ESCs transiently transfected with the FUCCI reported mAG-hGem. (**E**) ESCs expressing the FUCCI reporter gene were analysed by flow cytometry. The histogram shows the proportion of cells and fluorescence intensity. The DNA content for each gated population is indicated. Green negative population (blue marked) are cells in G1 phase, low-medium intensity green positive population (orange marked) are cells in S phase, and high intensity green positive population (green marked) are cells in late S/G2/M phase. (**F**) ESCs expressing the FUCCI reporter gene were isolated by FACS and total cell extracts were analysed by western blot with the indicated antibodies.

To investigate whether histone deacetylation and methylation are functionally linked after fork passage in ESCs, we used valproic acid (VPA), a specific inhibitor of class I HDACs with high affinity for HDAC1 (39). VPA holds promise in regenerative medicine, since it has shown to be a potent inducer of pluripotency from somatic cells (40,41). ESCs were treated with a short pulse (30 min) of VPA at low concentration with an EdU pulse during the last 10 min. Nascent chromatin was thereafter purified by iPOND and selected histone modifications were analysed by western blot with specific antibodies (Figure 6A). As expected, acute treatment with VPA led to a marked increase of H3K9Ac levels, both in the input and at nascent DNA (Figure 6A and B). Interestingly, H3K9 mono- and trimethylation was markedly reduced specifically on nascent DNA, in contrast to the input chromatin that remained unaffected. However, the other major repressive histone mark, the methylated lysine 27 at histone H3 and its acetylated form was not significantly affected. These results show that VPA has pronounced effects on the deposition of epigenetic marks during DNA replication, and suggests that in ESCs, HDAC1 could act at nascent DNA by regulating the rapid deacetylation of H3K9 in ESCs, which is necessary for their subsequent methylation during replication.

A rapid H3K9 deacetylation-coupled methylation mechanism is predicted to maintain stable levels of H3K9 modification during cell cycle progression. To test this hypothesis, we set up a system to isolate ESCs in different phases of the cell cycle. ESCs were stably transfected with the fluorescent cell cycle indicator human Geminin (amino acids 1–110) fused to the green fluorescent protein mAG1 (monomeric Azami-Green1) (FUCCI system) (42) (Figure 6C). This indicator is expressed in a cell cycle-dependent manner, being absent during G1 phase, gradually expressed in S phase and rapidly degraded at the end of M phase (Figure 6D) (42), allowing for the fluorescence-based separation of three populations of cells. ESCs in G1, S and late S/G2/M were prospectively isolated by fluorescence-activated cell sorting (FACS) (Figure 6E), and levels of H3K9Ac and HeK9me3 were assessed by western blot analysis. Unlike HeLa cells (36–38), no major alterations of H3K9me3 were observed when cells in G1 (green− ), S (green+) and late S/G2/M (green+++) cell cycle phases were compared (Figure 6F). Furthermore, H3K9Ac was largely unchanged with only a modest reduction in cells in late S/G2/M (green +++) (Figure 6D). These results suggest that, in contrast to tumour cells, the heterochromatin marker H3K9me3 is stably maintained across the cell cycle phases in ESCs.

## DISCUSSION

In this study we have profiled the proteins associated with the replication fork in ESCs with the assumption that ESCs contain in parts a unique protein interaction network and combined with our studies on their behaviour during DNA replication we conclude that: (i) Nascent DNA of ESCs is enriched with proteins associated with pluripotency; (ii) the recruitment at replication sites of proteins not belonging to the replisome, such as the MMR proteins and NuRD complex subunits, varies in a cell-type specific manner; (iii) complexes involved in determining the unique epigenetic landscape of ESCs, including the HDAC1–NuRD complex, are dynamically associated with chromatin during replication progression; and (iv) restoration of the repressive epigenetic mark H3K9me3 in ESCs is very rapid and coupled to a HDAC1-dependent deacetylation process.

Despite that two independent laboratories studied HEK-293T cells and used the same iPOND method (10,11), only 19 proteins were found in both studies from a total of 52 and 84 identified proteins, respectively. In the present study, 37 of 52 and 19 of 84 proteins identified in (10) and (11), respectively, were also found in our data (Supplementary Table S1). The differences between the studies could be caused by variations of the iPOND methodology, the amount of cells used in the studies (3.5 × 10^9^ by Fernandez-Capetillo group, 2.7 × 10^8^ by Cortez group and 3 × 10^7^ in the present study) and/or differences associated with the cell type studied (HEK-293T versus ESCs). Limitations in sensitivity resulting in false negative as well as unspecific noise leading to false positive might also contribute. Nevertheless, among the proteins identified, 75% of the replisome proteins and 60% of the DNA repair proteins identified in ESCs were common also to HEK-293T cells (10,11). These include among others the catalytic subunit and primase (POLA1 and PRIM2) from the polymerase alpha complex, which initiates the DNA synthesis; subunits of the DNA polymerases delta and epsilon (POLD1, D3 and E), which extend the synthesis of DNA at leading and lagging strand; the clamp loader complex replication factor C replication factor complex (RFC1–5); the subunits of the the CAF1 (CHAF1A and B) and FACT (SUPT16h, SSRP1) histone chaperon complex; the MCM helicase complex (MCM2, 3, 4, 6 and 7); and mismatch repair proteins (MSH2, 3 and 6). Considering that our and these previous studies on HEK293 cells are based on an unbiased method, which covers all active DNA replication from early to late S phase, and on a hypothesis-free proteomic approach, the high overlap in functional clusters confirms the existence of a core of replication associated proteins shared between different cell types. In addition to shared core proteins with HEK293, our side-by-side comparison of ESCs and fibroblasts suggest the existence of variable modules that associate with DNA in a cell-type dependent manner, like the NuRD complex. We believe that further validations of candidate proteins in different cell types will clarify the common and unique proteins at nascent DNA.

While the iPOND technique opens for an unbiased identification of the proteome associating with replicating DNA, some limitations should be considered. The iPOND technique fails to capture the dynamics of DNA replication along the S phase, and therefore it is not possible to ascertain that all the proteins identified in our or other studies are present at replication sites at the same time. However, a combination of iPOND with preparative flow cytometry techniques would allow for studies of the dynamics of the proteome around nascent DNA at selected stages of the S phase. Moreover, the identification of proteins associated with nascent DNA by iPOND does not necessarily evidence a physical interaction between them. The use of alternative methods, such as Förster Resonance Energy Transfer (FRET), or adaptations of iPOND such as performed in our two-step purification procedure (IP-iPOND), will be crucial as a systematic strategy to confirm coexistence of different components at the same nascent DNA.

Our study identified a large number of proteins associated with nascent DNA in ESCs that encompasses different functional clusters. As expected, the DNA replication cluster was enriched in our data set as well as an unexpected presence of a number of other functional clusters, including metabolic enzymes, ribosomal proteins and structural proteins. Although more experimental data is required to independently confirm the presence of these proteins at nascent DNA in ESCs, previous work has already pointed out to a possible functional relationship with DNA replication. For instance, the metabolic cluster includes enzymes of the mevalonate pathway, such as phosphomevalonate kinase and hydroxymethylglutaryl-CoA synthase. The inhibition of the mevalonate pathway by statins is known to cause a rapid block of DNA synthesis both in transformed cell lines and ESCs, which is followed by a loss of pluripotency (43,44). These effects are reversed by the addition of mevalonate, however, the mechanism of action of statins on DNA replication and the molecular mechanism by which mevalonate reverses these effects are unknown. Our results showing an interaction of these enzymes at the nascent DNA warrant for further studies identifying their role for maintenance of pluripotency across the cell cycle. Ribosomal proteins have previously been shown to have functional and physical association with DNA replication proteins in bacteria (45,46). In mammals, the ribosomal protein S27L is recruited to DNA breaks upon DNA damage and modulates the DNA damage response in human colorectal cancer cells (47). Our finding of the ribosomal protein S27L at nascent DNA and the close relation of DNA repair response pathway and DNA replication support a putative functional role of this protein for genome stability during replication in ESCs. Furthermore, biochemical studies in HeLa cells indicate that the lamina-associated polypeptide 2 isoform beta, an inner nuclear membrane protein and seemingly other splicing isoforms, including the alpha isoform identified in our study, regulate the initiation of DNA replication (48). Hence we have identified a connection between DNA replication with disparate unexpected cellular functions that opens for future studies resolving their functional roles in the replication machinery.

Although epigenetic modifications in ESCs are highly specific and underlie the plasticity necessary for gene expression to support development while retaining pluripotency (1), little is known on whether genetic structures selected by chromatin modifying enzymes are targeted during replication and/or in mature chromatin (49). Here, we show that HDAC1–NuRD complexes are enriched at chromatin during replication in ESCs. Moreover, we show that, in addition to HDAC1, the NuRD subunit CHD4 associates with UHRF1. These results point to a previously unidentified multi-enzyme complex ensuring epigenetic memory in ESCs. The abundance and versatile nature of protein complexes at nascent DNA identified in the present study reveals the importance of replication-associated protein complexes for establishment and maintenance of stem cell characteristics in ESCs.

The sequential recruitment of chromatin modifying enzymes often reflects a temporal ordered modification of chromatin. For instance, the observed interdependence between NuRD and PRC2 to propagate epigenetic marks (7,50,51) suggests a hierarchical relationship, by which deacetylation of H3K27Ac by NuRD is required to recruit PRC2 to the target sequences in order to methylate H3K27 (50,51). Similarly to H3K27 methylation, NuRD activity seems important for deacetylation of H3K9 that promotes methylation and the formation of silent chromatin containing H3K9me3 (51,52). Our finding of a H3K9 methylation associated to DNA replication is consistent with the identification of G9a/EHMT2, which associates with UHRF1 (34,53), and SETDB1 methyltransferases at the replication fork in our and other studies (54,55). The failure of H3K9 methylation during replication after VPA treatment suggests that licensing by deacetylation precedes and is required for methylation and the formation of silenced chromatin. Hence in contrast to somatic cells, our results support a mechanism for restoring H3K9me3 in ESCs that is very rapid, and which could participate in preventing the unscheduled expression of repressed chromatin.

Finally, we envisage that our results showing the existence of cell-type variations in replication associated proteins across different types of cells will encourage new directions of research in other fields, including cancer biology. Tumour and normal cells display different behaviour in the DNA repair response (56) and furthermore it is possible that changes in replication coupled mechanisms necessary for successful fork progression participate in therapy resistance and therapy-driven evolution of tumour recurrence, as exemplified recently for instance in glioma (57,58). An identification of mechanism participating in tumour evolution and therapy resistance could open for the identification of new targets for cancer therapy.

## SUPPLEMENTARY DATA

Supplemental data are available at NAR Online including [1–6].

SUPPLEMENTARY DATA
