# Supplementary material for: Identification of a large protein network involved in epigenetic transmission in replicating DNA of embryonic stem cells
Source: Nucleic Acids Res. 2014 May 22;42(11):6972–86. doi: 10.1093/nar/gku374 (PMC4066787; doi:10.1093/nar/gku374)
Supplement: SUPPLEMENTARY DATA [file supp_42_11_6972__index.html]

Identification of a large protein network involved in epigenetic transmission in replicating DNA of embryonic stem cells — SUPPLEMENTARY DATA 

# Identification of a large protein network involved in epigenetic transmission in replicating DNA of embryonic stem cells

## SUPPLEMENTARY DATA

**Files in this Data Supplement:**

- SUPPLEMENTARY DATA
- SUPPLEMENTARY DATA
- SUPPLEMENTARY DATA
