# Supplementary material for: Identification of a large protein network involved in epigenetic transmission in replicating DNA of embryonic stem cells
Source: Nucleic Acids Res. 2014 May 22;42(11):6972–86. doi: 10.1093/nar/gku374 (PMC4066787; doi:10.1093/nar/gku374)
Supplement: SUPPLEMENTARY DATA [file supp_gku374_nar-00461-h-2014-File009.pdf]

Supplemental Information

Identification of a large protein network involved in DNA fidelity and epigenetic transmission in replicating DNA of embryonic stem cells

Sergi Aranda, Dorothea Rutishauser and Patrik Ernfors

INVENTORY OF SUPPLEMENTAL INFORMATION

SUPPLEMENTAL DATA

Figure S1, related to Figure 1.

Figure S2, related to Figure 1.

Figure S3, related to Figure 2.

Figure S4, related to Figure 2.

Figure S5, related to Figure 4.

Table S1, Master Table for iPOND-MS in ESCs. (Available as Excel file)

Table S2, HDAC1 interacting mass spectrometry data. (Available as Excel file)

Table S3, List of antibodies used in this study

SUPPLEMENTAL MATERIALS AND METHODS

SUPPLEMENTAL REFERENCES

SUPPLEMENTAL DATA

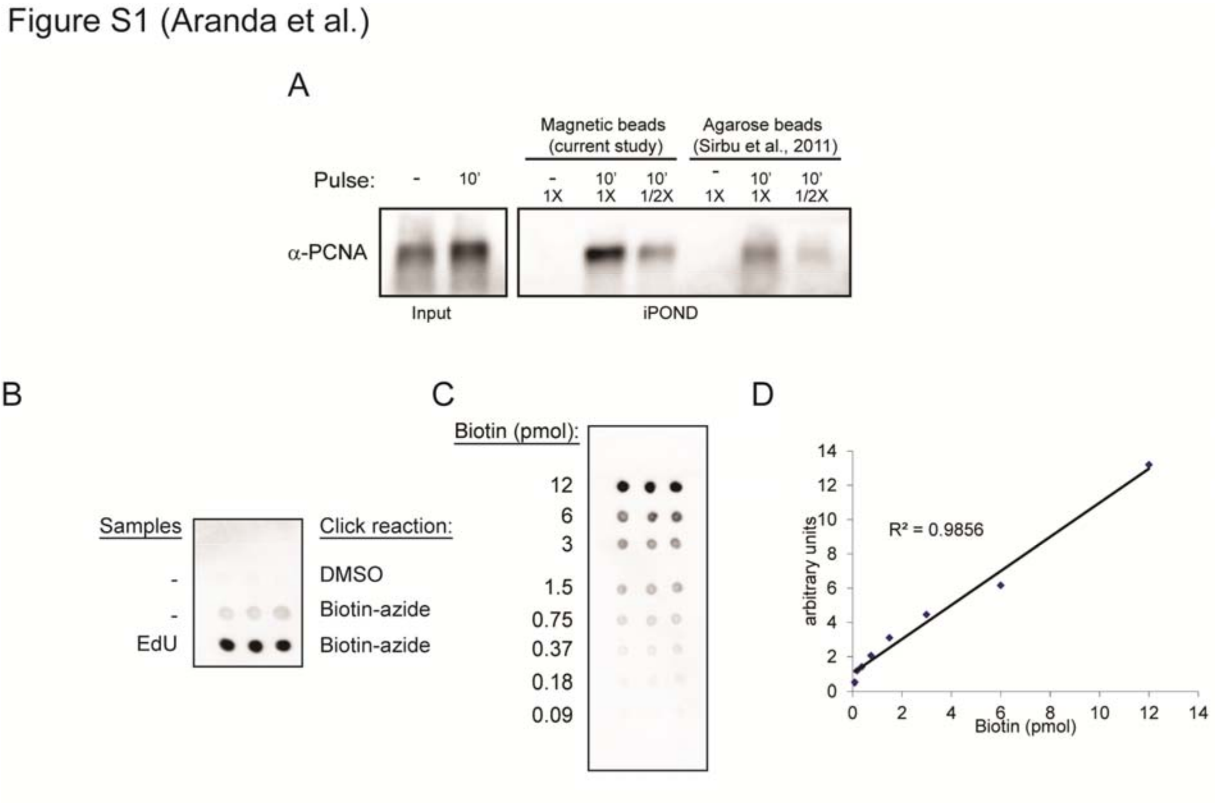

Figure S1. Related to Figure 1. (**A**) Comparison of protein isolation on nascent DNA using magnetic beads or agarose beads. Input (Inp) and the isolated proteins on nascent DNA (iPOND), using either magnetic or agarose streptavidin beads, from ESCs non-pulsed (-, control) and pulsed 10 minutes with EdU were analysed by Western blot using an antibody against PCNA. Note the greater efficiency of protein isolation by using magnetic beads. (**B**) Non-labelled and EdU-labelled ESCs were processed as indicated in the iPOND procedure. To assess the incorporation of biotin in the samples, sonicated samples were dot blotted onto nitrocellulose membrane in triplicates and incubated with HRP-streptavidin. (**C**) Two-fold serial dilutions of a biotinylated oligonucleotide were dot blotted onto nitrocellulose membrane in triplicates and hybridized with HRP-streptavidin. (**D**) The graph shows the quantification for the two-fold serial dilutions of the biotinylated oligonucleotide shown in C.

Figure S2 (Aranda et al.)

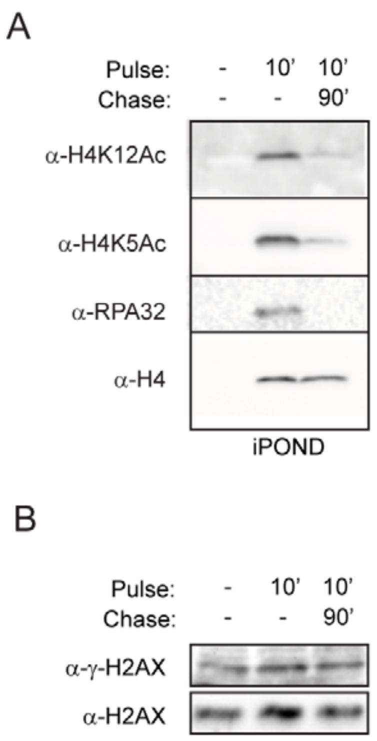

Figure S2. Related to Figure 1. (**A**) ESCs were incubated with EdU as indicated. The iPOND samples were analysed by Western blot using the indicated antibodies. (**B**) ESCs were incubated with EdU as indicated. Total cell lysates were analysed by Western blot using the indicated antibodies.

Figure S3 (Aranda et al.)

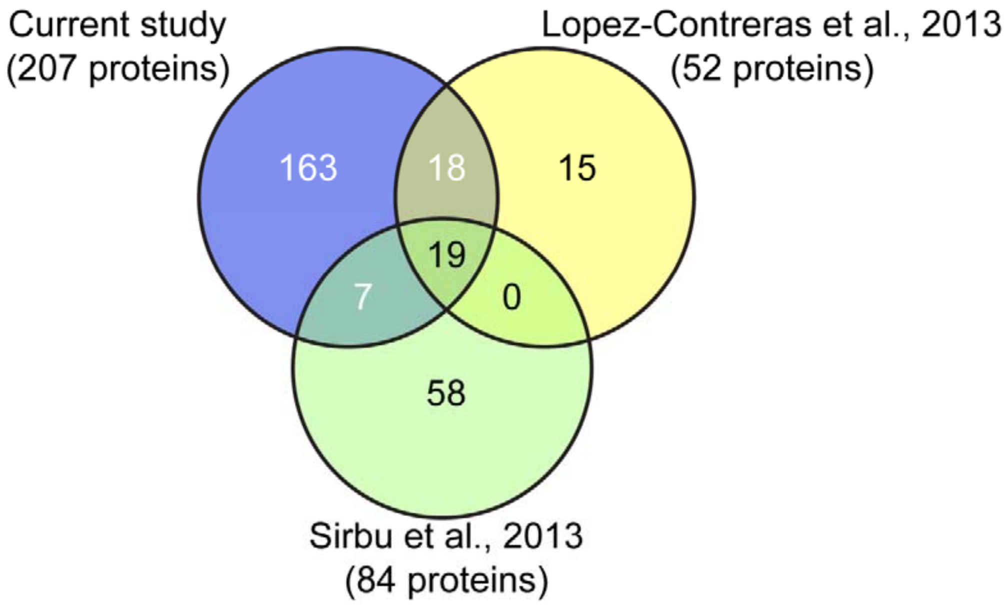

Figure S3. Related to Figure 2.Venn diagram indicating the number of proteins overlapping among the data from the current study, the data from Fernandez-Capetillo's group (Lopez-Conteras et al., 2013, ref#1) and the data from Cortez's group (Sirbu et al., 2013, ref#2). The total number of proteins identified in each study by iPOND at nascent DNA is indicated in brackets.

Figure S4 (Aranda et al.)

Replisome			
**Symbol**	**Protein Name**	**ESCs**	**NIH3T3**
**DNA primase**			
Pola1	DNA polymerase alpha catalytic subunit	+	+
Pola2	DNA polymerase alpha subunit B	+	+
Prim2	DNA primase large subunit	+	+
**DNA polymerases**			
Pold1	DNA polymerase delta catalytic subunit	+	+
Pold2	DNA polymerase delta subunit 2	+	
Pold3	DNA polymerase delta subunit 3	+	
Pole	DNA polymerase epsilon catalytic subunit A	+	+
**Clamp loader complex**			
Rfc1	Replication factor C subunit 1	+	+
Rfc2	Replication factor C subunit 2	+	+
Rfc3	Replication factor C subunit 3	+	+
Rfc4	Replication factor C subunit 4	+	+
Rfc5	Replication factor C subunit 5	+	+
**Replisome associated proteins**			
Lig1	DNA ligase 1	+	+
Lig3	DNA ligase 3	+	
Paf	PCNA-associated factor	+	
Timeless	Protein timeless homolog	+	
Fen1	Flap endonuclease 1	+	
Gins1	DNA replication complex GINS protein PSF1	+	
Wdhd1/And1	WD repeat and HMG-box DNA-binding protein 1	+	
Rpa1	Replication protein A 70 kDa DNA-binding subunit	+	+
Top2b	DNA topoisomerase 2-beta	+	+
Pcna	Proliferating cell nuclear antigen	+	+
**MCM complex**			
Mcm2	DNA replication licensing factor MCM2	+	+
Mcm3	DNA replication licensing factor MCM3	+	+
Mcm4	DNA replication licensing factor MCM4	+	
Mcm6	DNA replication licensing factor MCM6	+	+
Mcm7	DNA replication licensing factor MCM7	+	+
**Histone chaperones**			
Chaf1a	Chromatin assembly factor 1 subunit A	+	
Chaf1b	Chromatin assembly factor 1 subunit B	+	+
Asf1	Histone chaperone ASF1A	+	
Supt16h	FACT complex subunit SPT16	+	+
Ssrp1	FACT complex subunit SSRP1	+	+
**Nucleoside diphosphate kinase**			
Nme1	Nucleoside diphosphate kinase A	+	
Nme2	Nucleoside diphosphate kinase B	+	+

Figure S4. Related to Figure 2**.** Proteins identified in the iPOND proteomic analysis in ESCs (red) and fibroblasts (NIH3T3, green) that belongs to the core replisome module. + indicates presence of the protein.

Figure S5 (Aranda et al.)

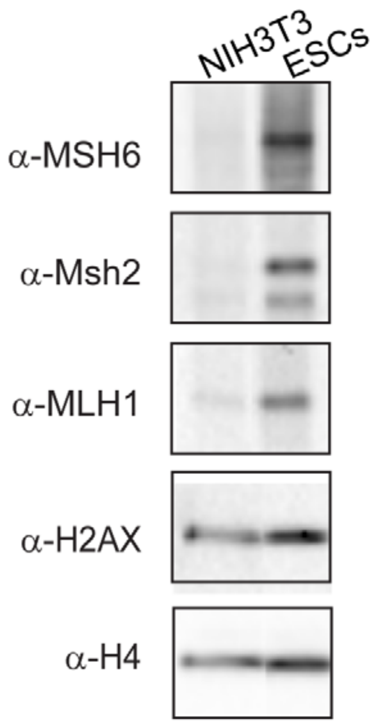

Figure S5. Related to Figure 3**.** Cell extracts from the fibroblast cell line NIH3T3 and ESCs were analysed by Western blot with the indicated antibodies.

Figure S6 (Aranda et al.)

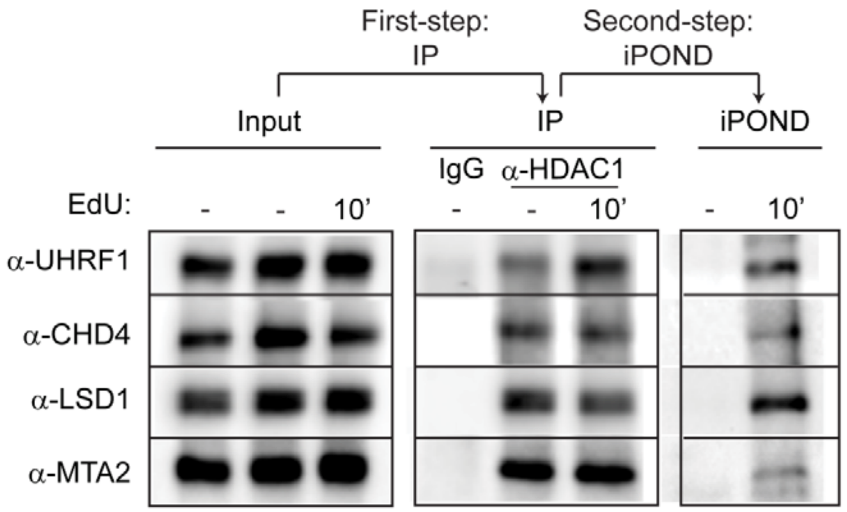

Figure S6. Related to Figure 4. Extracts from ESCs pulsed with EdU were immunoprecipitated with control immunoglobulins (IgG) or an anti-HDAC1 antibody (First -step: IP). The precipitates were eluted with a competitor peptide and re-purified using streptavidin magnetic beads (Second-step: iPOND). Samples were analysed by Western blot with the indicated antibodies.

Figure S7 (Aranda et al.)

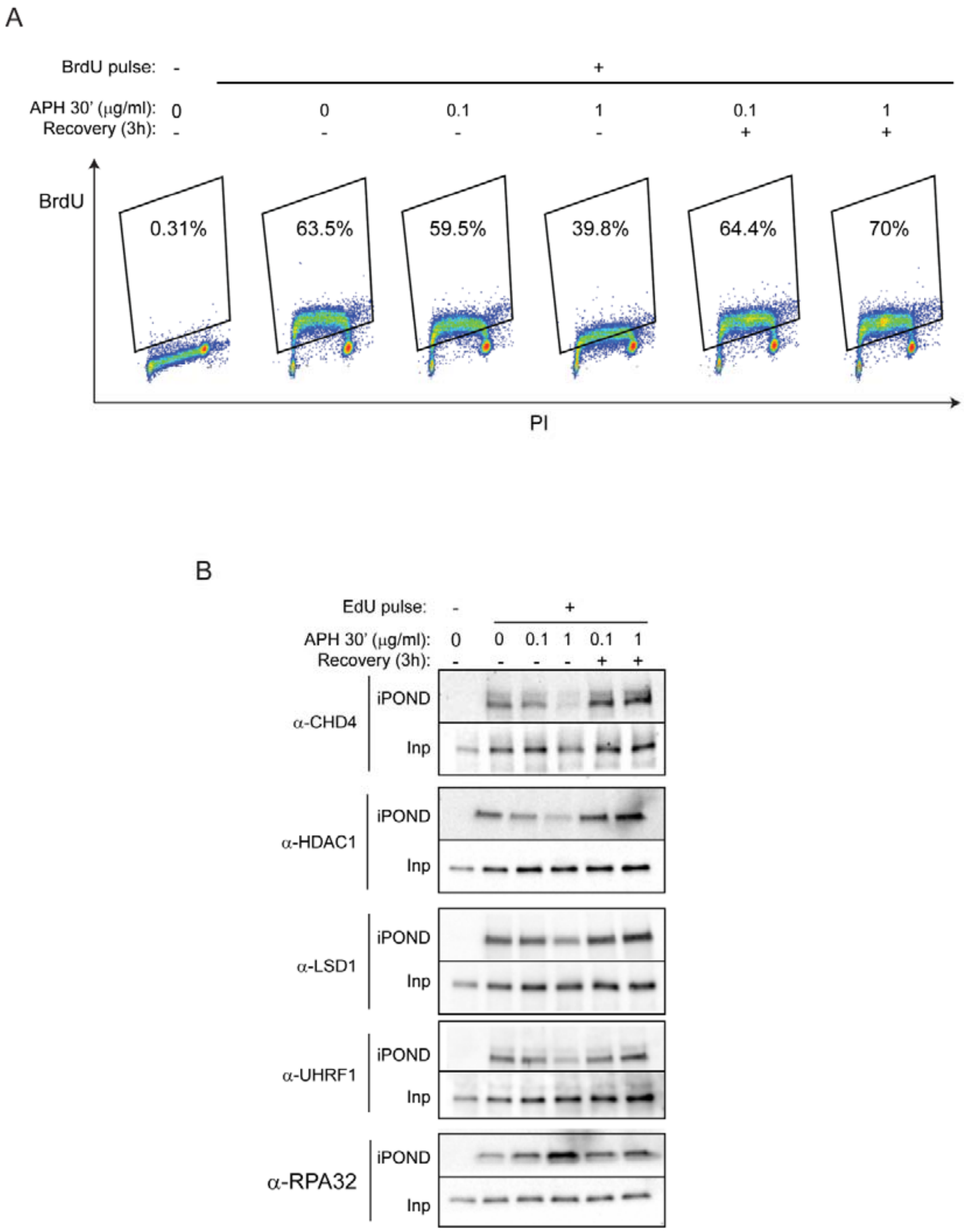

Figure S7. Related to Figure 4. (**A**) ESCs were treated 30 minutes with 0.1 or 1 μg/ml of aphidicolin (APH) as indicated. After the treatment, aphidicolin was removed from the media,

cells were washed with fresh media and incubated for additional 3 hours (Recovery). At the end of each treatment ESCs were pulsed for 10 minutes with BrdU. Cells were stained with propidium iodide and an FITC conjugated anti-BrdU antibody and were analysed by flow cytometry. Numbers indicate the percent of cells gated as BrdU positive cells in S-phase. Note the reduction of BrdU incorporation after the aphidicolin treatment and the recovery after aphidicolin removal. (**B**) EdU normalized samples from ESCs treated as in (**A**), but pulsed with EdU, were used in iPOND and both inputs and iPOND samples were analyzed by Western blot with the indicated antibodies.

Table S3: List of antibodies used in the study.

Primary Antibody	Host	Source	Catalog No.	Usage	Dilution
FITC-BrdU	Mouse	BD Bioscience	556028	FACS	1:50
Nanog	Rabbit	Abcam	ab80892	IF	1:250
Nestin	Mouse	Abcam	ab6142	IF	1:500
Histone H2AX	Rabbit	Abcam	ab11175	WB	1:5,000
CHD4	Rabbit	Abcam	ab72418	WB	1:1,000
MTA2	Rabbit	Abcam	ab8106	WB	1:4,000
HDAC1	Mouse	Millipore	05-100	WB	1:1,000
HDAC1	Goat	Santa Cruz Biotechnology	sc-6298	IP	10 mg
UHRF1	Rabbit	Santa Cruz Biotechnology	sc-98817	WB/IP	1:1,000
LSD1	Rabbit	Abcam	ab17721	WB	1:1,000
PCNA	Rabbit	Abcam	ab18197	WB	1:2,000
RPA32	Rat	Cell Signaling	2208	WB/IF	1:2,000/1:250
MLH1	Rabbit	Epitomics	2786-1	WB	1:1,000
MSH2	Rabbit	Epitomics	2848-1	WB	1:1,000
MSH6	Rabbit	Epitomics	2846-1	WB	1:1,000
Histone H3	Rabbit	Abcam	ab1791	WB	1:20,000
Histone H4	Rabbit	Millipore	05-858	WB	1:10,000
H4K12Ac	Rabbit	Millipore	07-595	WB	1:5,000
H4K5Ac	Rabbit	Millipore	07-327	WB	1:5,000
H3K9Ac	Rabbit	Cell Signaling	9671	WB	1:1,000
H3K9me1	Rabbit	Millipore	07-450	WB	1:1,000
H3K9me3	Rabbit	Abcam	ab8898	WB	1:2,000
H3K4Ac	Rabbit	Active Motif	39382	WB	1:1,000
H3K4me3	Rabbit	Diagenode	pAb-003	WB	1:1,000
H3K27Ac	Rabbit	Active Motif	39136	WB/ChIP	1:1,000
H3K27me3	Rabbit	Millipore	07-449	WB	1:1,000
gamma-H2AX	Mouse	Abcam	ab22551	IF/FACS	1:1,000
GAPDH	Mouse	SIGMA	G8795	WB	1:100,000
**Secondary Antibody**					
Anti-rabbit IgG (Conformation Specific)	Mouse	Cell signaling	3678	WB	1:1,000
HRP anti-Mouse IgG	Goat	SIGMA	A-4416	WB	1:2,000
HRP anti-Rabbit IgG	Goat	SIGMA	A-6667	WB	1:2,000
HRP anti-Rat IgG	Goat	SIGMA	A-9037	WB	1:2,000
anti-mouse alexa 555	Donkey	Molecular Probes	A31570	IF	1:400
anti-rat alexa 488	Donkey	Molecular Probes	A-21208	IF	1:400

SUPPLEMENTAL MATERIALS AND METHODS

FACS

Cells were harvested, fixed with ethanol and stained with a mouse fluorescein isothiocyanate (FITC)-conjugated anti-5-bromo-2'-deoxyuridine (BrdU) antibody. DNA was stained with propidium iodide (Sigma). The cells were sorted using FACScan (Becton Dickinson), and the data were analysed using FlowJo 7.5.5.

Immunofluorescence

The cells were pulsed for 10 minutes with 10 μM EdU (Invitrogen), then fixed in 4% paraformaldehyde for 10 min, incubated 30 min in blocking buffer (1% bovine serum albumin, 10% normal donkey serum, 0.1 % Triton X-100 in PBS). Then cells were stained with mouse anti-Nanog and rabbit anti-Nestin antibodies. Donkey Alexa-488 anti-mouse and Alexa-555 anti-rabbit were used as secondary antibodies. Click reaction (100 mM Tris-HCl [pH 8], 2 mM $CuSO_4$, 2 μM 647 Alexa-azide [Invitrogen] and 100 mM $C_6H_8O_6$ [ascorbic acid]) was performed for 30 min at RT. Samples were analysed using an Olympus FV1000 Confocal microscope, and optical sections were captured.

Dot-blot analysis

The samples were spotted in 1-μl dots onto a nylon membrane (Hybond-N+, Amersham) in triplicate, air-dried, and cross-linked (Stratalinker 1800, Stratagene, 0.4 J/cm^2). Membranes were incubated with horseradish peroxidase (HRP)-conjugated Avidin (1:1000, Invitrogen) for 30 min at RT. The data were quantified using Image Lab software (v4.0.1; Bio-Rad). A serially diluted 5'-biotinylated oligonucleotide served as standard (5'-CTCATAGCTCACGCTGTAGGTATCTCAGTTCGG-3').

DNA purification

The sonicated samples were reverse cross-linked by dilution in digestion buffer (250 mM NaCl and 50 ng/ml RNase A) overnight at 65°C and digested with proteinase K (0.1 mg/ml). The DNA was recovered by phenol/chloroform extraction and ethanol precipitation.

Immunoprecipitation in the presence of ethidium bromide

Cells were processed using the two-step (IP-iPOND) purification procedure, and 100 μg/ml ethidium bromide was added to the sonicated material, followed by incubation for 30 min at 4°C. The supernatant was collected by centrifugation (15,000x*g*, 20 min at 4°C) and incubated with 3 μg of either a rabbit anti-UHRF1 or rabbit IgG immunoglobulins overnight at 4°C. The immunocomplexes were incubated 1 h at RT with 25 μl Dynabeads Protein A (Invitrogen), washed 3 times with PBS + 2 mg/ml single strand DNA (SIGMA), and resuspended in Laemmli buffer.

In-gel digestion of silver-stained gel bands

The iPOND samples were resolved by SDS-PAGE, and the gels were silver-stained (Pierce). Four independent isolations of nascent chromatin from ESCs, with their corresponding negative control, and three isolations of mature chromatin after a 90-min chase were performed (Experiment I, II, III, IV). Two independent nascent chromatin isolations were performed from NIH3T3 fibroblasts cells (Experiment V and VI). Each gel lane was divided into 24 bands, which were de-stained in 50 mM ammonium bicarbonate and 50% acetonitrile. Tryptic digestion was performed by a liquid-handling robot (MultiProbe II, Perkin Elmer), including protein reduction in 10 mM DTT and alkylation in 55 mM iodacetamide. Gel pieces were dehydrated in 100% acetonitrile, trypsin was added to a final concentration of 13 ng/μl, and the pieces were digested for 5 h at 37°C. Extracted peptides from consecutive bands were pooled according to their protein levels, resulting in eight pools for each lane.

Liquid chromatography tandem mass spectrometry

Samples from experiment I were analyzed using a Q-TOF Premier API instrument (Water, Milford, MA, USA) coupled to a Waters NanoAquity UPLC. Peptides were first desalted for 1 min on a Symmetry C18 trapping column (5 μm, 180 μm x 2 cm) and separated on an analytical BEH C18 column (1.7 μm, 75 μm i.d. x 15 cm). Peptides were eluted with a linear 90 min gradient from 3-40% ACN at a flow rate of 300 nl/min. Data was acquired in data-dependent mode over a mass range of m/z 300 to m/z 2000 by automated switching between MS and MS/MS. The collision energy was altered between 25 and 45 eV depending on the mass and

charge state. Scan speed was 0.9 s/spectra with 0.1 interscan time and lock mass reference was applied (Glu fribrinogen B, Sigma).

Nano-LC-MS/MS analyses from experiment II to VI were performed using an Easy-nLC system (Thermo Scientific) directly coupled to a hybrid LTQ Orbitrap Velos ETD mass spectrometer (Thermo Scientific). Peptides were separated in a 10-cm fused SilicaTip column (New Objective, Inc.) that was packed in-house with 3-µm C18-AQ ReproSil-Pur (Dr. Maisch GmbH) using a linear gradient from 3−48% acetonitrile in 89 min at a flow rate of 300 nl/min. The MS acquisition method was comprised of one survey full scan ranging from m/z 300 to m/z 2000 acquired in the FT-Orbitrap with a resolution of R= 60‘000 at m/z 400, followed by up to five data-dependent HCD MS2 scans from the top five precursor ions with a charge state ≥ 2 and at R=7500. For the HDAC1-centered protein network experiment the MS acquisition method was comprised of the same survey full scan and up to ten data-dependent CID MS2 scans in profile mode from the top ten precursor ions with charge state ≥ 2.

Database searches

Tandem mass spectra were extracted using Raw2MGF (in-house software), and the resulting mascot generic files from each lane were searched against the mouse SwissProt protein database using the Mascot Deamon 2.3.0 search engine (Matrix Science Ltd.), which was set to search the SwissProt protein database (selected for Mus musculus, version 2011.08) using trypsin and two missed cleavage sites. Peptide mass tolerance was set to 10 ppm and 0.05 Da for the fragment ions. Carbamidomethylation of cysteine was specified as a fixed modification, whereas oxidation of methionine and deamidation of asparagine and glutamine were defined as variable modifications. The lists of identified proteins were exported from the .dat files using the following criteria: significance threshold of 0.05, MudPit protein scoring, required red bold, and including same-set proteins. A red bold match is the highest scoring match to a particular query listed under the highest scoring protein containing that match.

The following criteria were used for inclusion in Supplementary Table S1 as nascent DNA bound proteins or mature DNA bound proteins: proteins present with at least one significant

protein sequence in two out of four independent experiments (two out of two in the case of NIH3T3 cells) and with a Mascot score at least 3-fold higher than the corresponding control and the corresponding pulse-chase experiment processed in parallel were considered enriched at nascent DNA. Proteins with a Mascot score at least 3-fold higher in pulse-chase than the corresponding pulse experiments were considered enriched at mature DNA.

System-wide analyses

Identifiers for human orthologues were retrieved using the biological DataBase network (3). The pair-wise analysis was performed on data from several genome-wide siRNA studies (see Figure Table 1 for references). Gene ontology term enrichment was performed using the DAVID database with the total set of proteins against the entire mouse genome as the background (4). Protein interaction data were retrieved from the STRING database (5) using the protein mode. Only interactions from protein-protein interaction databases, curated databases and text-mining information were considered (confidence score >0.4). The network was visualised using Cytoscape (v2.8.3) (6).

SUPPLEMENTAL REFERENCES

1. Lopez-Contreras, A.J., Ruppen, I., Nieto-Soler, M., Murga, M., Rodriguez-Acebes, S., Remeseiro, S., Rodrigo-Perez, S., Rojas, A.M., Mendez, J., Munoz, J. *et al.* (2013) A proteomic characterization of factors enriched at nascent DNA molecules. *Cell Rep*, **3**, 1105-1116.

2. Sirbu, B.M., McDonald, W.H., Dungrawala, H., Badu-Nkansah, A., Kavanaugh, G.M., Chen, Y., Tabb, D.L. and Cortez, D. (2013) Identification of Proteins at Active, Stalled, and Collapsed Replication Forks Using Isolation of Proteins on Nascent DNA (iPOND) Coupled with Mass Spectrometry. *J Biol Chem*, **288**, 31458-31467.

3. Mudunuri, U., Che, A., Yi, M. and Stephens, R.M. (2009) bioDBnet: the biological database network. *Bioinformatics*, **25**, 555-556.

4. Huang da, W., Sherman, B.T. and Lempicki, R.A. (2009) Systematic and integrative analysis of large gene lists using DAVID bioinformatics resources. *Nat Protoc*, **4**, 44-57.

5. Jensen, L.J., Kuhn, M., Stark, M., Chaffron, S., Creevey, C., Muller, J., Doerks, T., Julien, P., Roth, A., Simonovic, M. *et al.* (2009) STRING 8--a global view on proteins and their functional interactions in 630 organisms. *Nucleic Acids Res*, **37**, D412-416.

6. Smoot, M.E., Ono, K., Ruscheinski, J., Wang, P.L. and Ideker, T. (2011) Cytoscape 2.8: new features for data integration and network visualization. *Bioinformatics*, **27**, 431-432.
